# Predicting short-term suicidal thoughts in adolescents using machine learning: developing decision tools to identify daily level risk after hospitalization

**DOI:** 10.1017/S0033291721005006

**Published:** 2023-05

**Authors:** E. K. Czyz, H. J. Koo, N. Al-Dajani, C. A. King, I. Nahum-Shani

**Affiliations:** 1 Department of Psychiatry, University of Michigan, Ann Arbor, MI, USA; 2 Institute for Social Research, University of Michigan, Ann Arbor, MI, USA

**Keywords:** Adolescents, daily diary, ecological momentary assessment, just-in-time adaptive intervention, machine learning, risk algorithm, suicidal ideation

## Abstract

**Background:**

Mobile technology offers unique opportunities for monitoring short-term suicide risk in daily life. In this study of suicidal adolescent inpatients, theoretically informed risk factors were assessed daily following discharge to predict near-term suicidal ideation and inform decision algorithms for identifying elevations in daily level risk, with implications for real-time suicide-focused interventions.

**Methods:**

Adolescents (*N* = 78; 67.9% female) completed brief surveys texted daily for 4 weeks after discharge (*n* = 1621 observations). Using multi-level classification and regression trees (CARTSs) with repeated 5-fold cross-validation, we tested (a) a simple prediction model incorporating previous-day scores for each of 10 risk factors, and (b) a more complex model incorporating, for each of these factors, a time-varying person-specific mean over prior days together with deviation from that mean. Models also incorporated missingness and contextual (study week, day of the week) indicators. The outcome was the presence/absence of next-day suicidal ideation.

**Results:**

The best-performing model (cross-validated AUC = 0.86) was a complex model that included ideation duration, hopelessness, burdensomeness, and self-efficacy to refrain from suicidal action. An equivalent model that excluded ideation duration had acceptable overall performance (cross-validated AUC = 0.78). Models incorporating only previous-day scores, with and without ideation duration (cross-validated AUC of 0.82 and 0.75, respectively), showed relatively weaker performance.

**Conclusions:**

Results suggest that specific combinations of dynamic risk factors assessed in adolescents' daily life have promising utility in predicting next-day suicidal thoughts. Findings represent an important step in the development of decision tools identifying short-term risk as well as guiding timely interventions sensitive to proximal elevations in suicide risk in daily life.

The prevention of suicide deaths and related outcomes in youth, including non-lethal attempts and suicidal thoughts, is an urgent public health priority. The prevalence of suicide deaths among adolescents in the United States has been on the rise, showing a nearly 60% increase between 2007 and 2018 (Curtin, 2020). Periods of high-risk transition, such as following psychiatric hospitalization, are associated with particularly elevated suicide risk (Chung et al., 2019). Approximately 20% of discharged youth experience a suicide-related event (e.g. rehospitalization, suicide attempt) within 3 months and between 30% and 40% experience these events within 6 months after discharge (Czyz, Berona, & King, 2016a, 2016b; Kennard et al., 2018; Yen et al., 2013, 2019). Discharged adolescents are also vulnerable to suicidal thoughts of varying intensity and chronicity (Prinstein et al., 2008; Wolff et al., 2018), with more persistent suicidal ideation patterns showing an especially strong association with suicide attempts (Czyz & King, 2015; Prinstein et al., 2008), and with prior research highlighting notable fluctuations in day-to-day suicidal thoughts shortly after hospitalization (Czyz, Horwitz, Arango, & King, 2018). Given that suicidal ideation and related outcomes are heterogeneous and time-varying, there is a critical need for strategies that can accurately detect elevations in suicidal ideation and risk more broadly as well as guide the timely delivery of interventions, particularly during high-risk periods.

## Identifying elevations in suicide risk in daily life

The pervasiveness of mobile phones has paved the way for use of intensive longitudinal assessment approaches – such as daily diaries (once-a-day assessments) and ecological momentary assessments (EMAs) characterized by repeated measurements within a day – that allow for sampling individuals' day-to-day experiences (Shiffman, Stone, & Hufford, 2008). By allowing for repeated and frequent assessment of experiences in real-world environments and over time, these approaches reduce recall bias and lead to more ecologically valid information about thoughts, behaviors, or feelings compared to traditional assessments spaced over longer time intervals (Shiffman et al., 2008). Daily diary and EMA studies are increasingly common in suicide prevention research (Davidson, Anestis, & Gutierrez, 2017; Gee, Han, Benassi, & Batterham, 2020; Kleiman & Nock, 2018), as they enable assessment of dynamic and short-term precursors of suicide risk. In addition to demonstrating the highly dynamic nature of suicidal thoughts (Hallensleben et al., 2017; Kleiman et al., 2017), this growing research has also examined short-term correlates and precipitants of suicidal ideation across different domains (e.g. situational, affective, interpersonal) among adults (Armey, Brick, Schatten, Nugent, & Miller, 2018; Ben-Zeev, Young, & Depp, 2012; Coppersmith, Kleiman, Glenn, Millner, & Nock, 2019; Hallensleben et al., 2019; Husky et al., 2017; Kleiman et al., 2017) and, to a lesser extent, among adolescents (Czyz et al., 2018; Nock, Prinstein, & Sterba, 2009). Of note, existing daily diary and EMA studies have largely examined individual indicators of suicidal ideation or relatively limited interactions guided by theory (Czyz et al., 2018; Hallensleben et al., 2019). A focus on individual predictors alone, or solely pre-specified interactions, may obscure important patterns in data that could be useful in detecting short-term risk. For example, a proof-of-concept study has shown that models that incorporate multiple risk and protective factors assessed daily after hospitalization predict near-term suicidal crises more accurately relative to models that incorporate single factors (Czyz, Yap, King, & Nahum-Shani, 2020).

In line with meta-analytic studies indicating that individual risk factors are only modestly predictive of suicidal thoughts and behavior (Franklin et al., 2016; Ribeiro et al., 2016), more research considering multiple risk factors (e.g. King et al., 2020) and their complex interactions may be needed to improve our understanding of real-time, real-world conditions that represent near-term suicide risk. Although not yet widely utilized in this context, data-driven approaches (such as machine learning) could aid in identifying such complex relationships, with implications for improving short-term risk detection and for guiding the delivery of timely support.

## Potential value of decision algorithms identifying short-term risk

Machine learning approaches (e.g. support vector machine, random forests, and artificial neural network) have been increasingly applied in suicide prevention research (see review Burke, Ammerman, & Jacobucci, 2019). These methods may be highly useful in identifying states of vulnerability representing elevations in suicide risk based on complex intensive longitudinal data by estimating or ‘learning’ parameters that can produce a reliable prediction of an adverse proximal outcome (e.g. near-term suicidal thoughts) (Nahum-Shani et al., 2018). These methods have the important advantage of handling large volumes of predictors and combining them in interactive and non-linear ways (Athey, 2017), yielding algorithmically optimized prediction models. Machine learning has shown promise in predicting suicidal ideation and behavior among adults (Chen et al., 2020; de la Garza, Blanco, Olfson, & Wall, 2021; Ribeiro, Huang, Fox, Walsh, & Linthicum, 2019; Walsh, Ribeiro, & Franklin, 2017) and adolescents (Hill, Oosterhoff, & Kaplow, 2017; Miché et al., 2020; Walsh, Ribeiro, & Franklin, 2018). Moreover, a study of adult inpatients responding to EMAs during hospitalization used machine learning models to predict suicide attempts 2–4 weeks later (Wang et al., 2021). To date, however, machine learning approaches have not been applied to predict near-term suicidal thoughts in daily life.

Applying such data-driven approaches, based on intensive longitudinal data, to detect proximal elevations in suicide risk may be especially valuable if it can aid in identifying opportune times to provide timely interventions that are sensitive to individuals' changing suicide risk levels in real-world conditions. For example, just-in-time adaptive interventions (JITAIs) use dynamically changing information about the individual's internal state and context to recommend whether, when, and how to deliver interventions in daily life using mobile technology (Nahum-Shani, Hekler, & Spruijt-Metz, 2015; Nahum-Shani et al., 2018). JITAIs employ decision rules that link dynamic information about the individual to specific intervention options, specifying the conditions in which intervention should be delivered. The goal is to provide the intervention option that is best for an individual at a given time point, while avoiding unnecessary intervention and burden (Nahum-Shani et al., 2015). Identifying states of vulnerability to an adverse proximal outcome (here, proximal suicidal thoughts) play a critical role in the formulation of effective JITAIs. Many JITAIs and similar ecological interventions focusing on mental health (see reviews: Balaskas, Schueller, Cox, & Doherty, 2021; Bidargaddi, Schrader, Klasnja, Licinio, & Murphy, 2020; Wang & Miller, 2020) are motivated to break the link between states of vulnerability (i.e. conditions that represent heightened risk) and a specific adverse proximal outcome via the delivery of timely intervention. However, to inform the development of real-time interventions for suicide prevention, more research is needed to empirically identify states of vulnerability or the conditions in which adolescents at risk for suicide may benefit from such interventions (i.e. *when* an intervention should be delivered). Hence, the current study seeks to leverage intensive longitudinal data and machine learning to arrive at a decision algorithm for detecting near-term suicidal thoughts, which, in turn, could be used to guide the formulation of real-time interventions (Nahum-Shani et al., 2015).

## The current study

In this daily diary study of psychiatrically hospitalized adolescents, a multi-level classification and regression tree (CART) was applied to predict proximally emerging suicidal ideation, operationalized as next-day suicidal ideation, during a high-risk post-discharge period. The multi-level CART was selected, instead of other machine learning strategies (e.g. random forest, elastic net, neural network), due to its ability to generate readily interpretable results (e.g. see Boudreaux et al., 2021 for an overview) while simultaneously considering intensively sampled predictors (i.e. multi-level data structure) and their complex combinations (Fokkema, Edbrooke-Childs, & Wolpert, 2020). Risk and protective factors were selected based on theoretical considerations and clinical relevance (Bandura, 1977; Klonsky & May, 2015; Rudd et al., 2006; Shneidman, 1993; Van Orden et al., 2010; Wenzel & Beck, 2008) and were assessed via electronic surveys each day over the course of 1 month after psychiatric hospitalization. The multi-level CART was used to arrive at a decision tool (i.e. algorithm) predicting the occurrence of next-day suicidal ideation. Two approaches were considered: (1) a simple prediction model incorporating previous-day scores for each individual factor, and (2) a more complex model incorporating, for each of these factors, a person-specific mean over the days prior together with the deviation (change score) from that person-specific mean. To the best of our knowledge, this is the first study that leveraged machine learning to generate decision algorithms for predicting short-term suicidal ideation in daily life. Such decision algorithms represent an important step toward the development of suicide-focused interventions addressing real-time, real-world changes in suicide risk.

## Methods

### Participants and procedures

Participants were psychiatrically hospitalized adolescents (ages 13–17) who were eligible to participate based on last-month suicide attempt and/or last-week suicidal ideation [with thoughts of either method, intent, and/or plan; based on the Columbia-Suicide-Severity Rating Scale (Posner et al., 2011)]). Exclusion criteria were severe cognitive impairment or altered mental status (psychosis, mania), transfer to a medical unit or residential placement, no availability of a legal guardian (ward of sate), or adolescents not having a cell phone. Described in detail elsewhere (Czyz et al., 2021), participants, were recruited between March 2019 and January 2020 as part of a psychosocial intervention pilot study focusing on feasibility and acceptability^†^^1^. Of those who provided study consent/assent (*n* = 82 or 87.2%), 80 completed baseline assessment and continued in the study. Adolescents completed daily surveys for 4 weeks, which were texted to their phones each evening beginning on the first day after discharge. Adolescents could respond to surveys between 5 and 8 pm and received $4 for completing each survey. This study's analytic sample includes 78 adolescents (97.5%) who completed at least two consecutive daily surveys; the survey completion rate was 74.2% (1621 out of 2184 possible daily surveys) over the 4-week period. On average, adolescents completed 20.78 daily surveys (s.d. = 6.92). The study's procedures complied with ethical standards for human subjects research and were approved by the participating university's Institutional Review Board.

### Measures

#### Daily outcome

Each day, adolescents responded to questions assessing the frequency of their suicidal thoughts in reference to the last 24 h (“How many times did you have thoughts of killing yourself?), with responses ranging from 0 (not at all) to 4 (all the time). Modeled after the Columbia-Suicide Severity Rating Scale (C-SSRS) (Posner et al., 2011), the item was previously adapted to assess daily suicidal ideation in adolescents (Czyz et al., 2018). A binary outcome was created to indicate the absence/presence (0 *v.* 1) of next-day suicidal ideation.

#### Daily predictors

Predictors were assessed with the following brief measures, adapted based on existing validated scales, to reduce response burden given that data were collected daily. Adolescents responded to all items in reference to the last 24 h.

*Hopelessness*. Participants rated the extent to which they felt hopeless (‘I see only bad things ahead of me, not good things’) using a 4-point scale from ‘strongly disagree’ to ‘strongly agree.’ This item was modeled after the 6-item Brief Hopelessness Scale (Bolland, McCallum, Lian, Bailey, & Rowan, 2001).

*Connectedness to Family and Friends*. Using a 7-point scale (from ‘not at all true for me’ to ‘very true for me’), adolescents rated the extent to which they felt close to their friends and, separately, to their family. These items were modeled after the Interpersonal Needs Questionnaire (INQ) (Van Orden, Cukrowicz, Witte, & Joiner, 2012), which measures thwarted belongingness and perceived burdensomeness. The INQ closeness item was adapted to reference a sense of closeness with peers (item 1) and with family (item 2).

*Burdensomeness*. Participants rated their perceived sense of burdensomeness (‘The people in my life would be happier without me’) on a 7-point scale ranging from ‘not at all true for me’ to ‘very true for me.’ This item was similarly based on the INQ (Van Orden et al., 2012)

*Agitation*. Participants rated their agitation (‘I felt so stirred up inside I wanted to scream’) on a 7-point scale. The item was modeled after an item from the Brief Agitation Measure (Ribeiro, Bender, Selby, Hames, & Joiner, 2011). The item phrasing was adjusted to reference experiences in the 24-h period and response options ranged from ‘not at all’ to ‘very much.’

*Worry and Rumination*. Using a scale ranging from 1 (‘not at all’) to 7 (‘very much’), participants reported levels of worry and rumination using two items. Rumination was assessed with the item ‘I was dwelling on my feelings and problems,’ and worry was assessed with the item ‘I was worried about things that could happen.’ These items were based on previous EMA studies of rumination and worry (Kircanski, Thompson, Sorenson, Sherdell, & Gotlib, 2015).

*Self-efficacy to Refrain from Suicidal Action*. Confidence to refrain from suicidal action (‘How confident are you that you will be able to keep yourself from attempting suicide?’) was assessed with an item from the 3-item Self-Assessed Expectations of Suicide Risk Scale (Czyz et al., 2016b), which showed predictive validity for suicide attempts in youth. Responses were rated from 0 (‘not at all confident’) to 10 (‘completely confident’).

*Psychological Pain*. Participants rated the extent to which they felt miserable as a proxy for psychological pain. Responses were rated on a 5-point scale (from ‘very slightly or not at all’ to ‘extremely’). This question was adapted from the 10-item positive and negative affect schedule for children (PANAS-C) (Ebesutani et al., 2012).

*Suicidal Ideation Duration*. Each day, adolescents reporting any suicidal ideation subsequently rated the duration of their suicidal thoughts (‘*How long did these thoughts last*?’) on a 5-point scale (from ‘a few seconds or minutes’ to ‘more than 8 h/continuous’). The duration of the ideation item was based on the C-SSRS (Posner et al., 2011). We created a continuous scale for ideation duration ranging from 0 (no ideation) to 5 (continuous ideation).

### Predictor preparation

For each of the 10 risk and protective factors of interest, we calculated the cumulative person-specific mean (each person's own mean) for each day and the deviation from that mean. Specifically, for each of the 10 factors, a person-specific mean was calculated for each day *t* based on daily responses up to and including day *t* (i.e. within-person sum of responses to the specific factor up to and including day *t*, divided by the number of completed surveys up to and including *t*). Additionally, for each factor, the deviation of day *t* response from the cumulative person-specific mean was calculated. The relatively simple prediction model included previous-day *t* responses for each of the 10 factors, whereas the more complex model included each factor's cumulative person-specific mean for each day *t* as well as day *t* deviation from that mean.

### Data analytic strategy

We fit a series of multi-level CART models to predict next-day suicidal ideation. Designed to accommodate multi-level and longitudinal data structures, these CART models employ generalized linear mixed model (GLMM) and decision trees; specifically, a GLMM tree algorithm uses an unbiased recursive partitioning method that selects the splitting variable based on the lowest *p* value until there are no more splitting predictors that are below our pre-specified *α* of 0.05 (Fokkema et al., 2020). A tree like-structure is produced using a process of partitioning data into subgroups such that observations in each group become more similar in terms of the outcome within groups, a process that is repeated until no additional improvements in classification of the outcome are identified based on available predictors. The CART has the advantage of not requiring any assumptions of the data and allowing for a large number of predictors to be included in the model. Moreover, CARTs provide easily interpretable results.

We initially fit two separate CART models: (a) a simple model including previous-day response for each of the 10 factors; and (2) a more complex model including, for each factor, the previous-day cumulative person-specific mean and deviation from that mean. The models also included indicators for a week in the study (ranging from week 1–4), day of the week (from Mon–Sun), and a variable indicating whether the previous-day survey was missing (previous-day missingness). To investigate the added value of measuring ideation duration as a predictive factor, we fit two additional models that were identical to the first two but excluded predictors based on ideation duration. The outcome across all models was the presence/absence of next-day suicidal ideation (binary outcome).

To evaluate model performance, we used blocked 5-fold cross-validation. This method includes random division of the data into five sets, such that four models are created and then tested on a single selected set. This process is repeated five times, with a different test set each time. Traditional random k-fold cross-validation methods do not consider dependent data structures (observations nested within individuals) and may thus provide too optimistic results, leading to incorrect confidence in predictions (Roberts et al., 2017). Also, unbalanced values of the outcome (i.e. higher proportion of zeros to ones) can be problematic for cross-validation (Thabtah, Hammoud, Kamalov, & Gonsalves, 2020). Thus, cross-validation was based on stratified blocked folds (i.e. individual participants serving as blocks) with similar prevalence of the outcome (Roberts et al., 2017). Folds were created such that all observations from a particular participant were within the same fold, and the proportion of high and low-risk individuals (based on the median split of the proportion of days ideation was endorsed) were kept consistent across the folds. The blocked 5-fold cross-validation process was repeated 10 items to improve stability and consistency of results. All the analyses were conducted in R version 4.0.2 (2020-06-22) utilizing glmertree, caret, pROC, and groupdata2 packages.

Model performance was evaluated using different metrics, including sensitivity, specificity, an area under the receiver operating characteristic curve (AUC), and positive predictive value (PPV). Sensitivity refers to the probability of identifying correctly those who would experience the outcome (i.e. next-day suicidal ideation) while specificity refers to the probability of identifying correctly those who would not experience the outcome. AUC captures the average sensitivity over all values of false-positive rates (i.e. 1-Specificity) of different cutoff points of predicted probability; AUC thus provides a measure of discriminatory ability (accuracy of differentiating cases with the outcome from those without), with values ranging from 0 to 1, where 0.5 indicates no discriminative ability (similar to chance) and 1 indicates perfect discrimination. Of note, to obtain maximal sensitivity and specificity for selected models, we sought to determine cutoffs on predicted probabilities using the ‘closest top left’ criterion implemented in the R package pROC (Robin et al., 2011). Finally, the PPV refers to the probability that those identified as having the outcome actually have the outcome (i.e. the proportion of true positives over the number of cases predicted to be positive based on the model). Unlike sensitivity and specificity, PPV is directly related to the prevalence rate of the outcome in the population; hence, PPV will increase with an increasing prevalence of the outcome in the population. Similar to AUC, sensitivity, specificity, and PPV values can range from 0 to 1, with values closer to 1 indicating better performance.

## Results

### Participant characteristics

Over 60% of participants were biological females (67.9%; *n* = 53), with a mean age of 15.19 (s.d. *=* 1.35) years. The distribution of race/ethnicity was as follows (more than one category could be selected): 65 (83.3%) White, five (6.4%) African American/Black, four (5.1%) Asian, four (5.1%) American Indian or Alaska Native, and one (1.3%) Native Hawaiian or Other Pacific Islander. Nine participants (11.5%) self-identified as Hispanic. At the time of hospitalization, all adolescents had last-week suicidal ideation, with a mean ideation rating of 3.90 (s.d. = 0.91) (range 0–5, where 0 is no ideation and 5 corresponds to ideation with intent and plan). In addition, half of the participants (*n* = 39) had at least one-lifetime suicide attempt. Over the course of 4 weeks after discharge, 64 (82.1%) participants reported a total of 631 instances of suicidal ideation (38.9%), out of 1621 completed daily surveys, with an average of 9.86 (s.d. = 7.91) instances. Descriptive information for the predictors and the outcome are provided in online Supplementary Table S1.

### Model performance

**Fig. 1.**
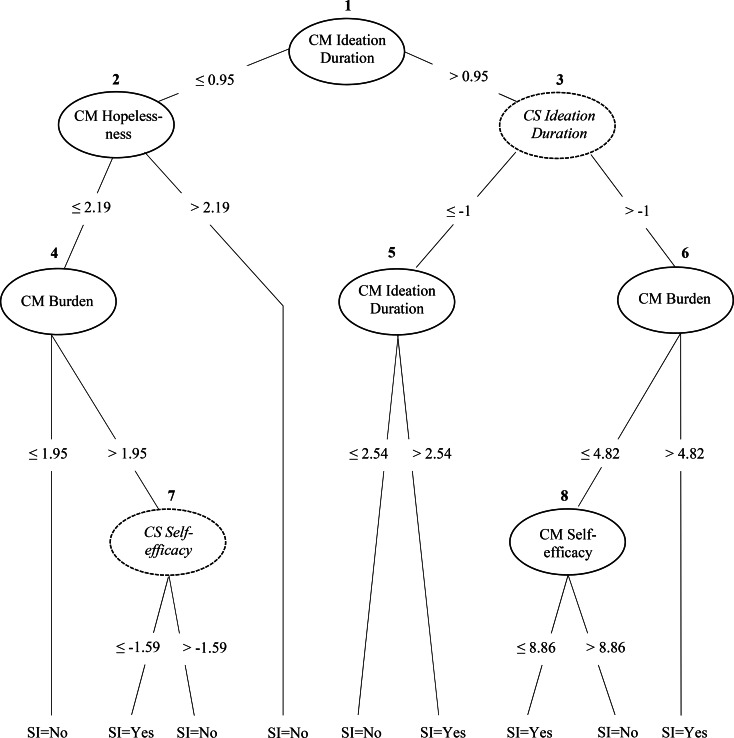
Prediction rule for next-day suicidal ideation. *Notes*: SI = Next-day suicidal ideation; CM = Cumulative person-specific mean; *CS* = Change score (deviation from the person-specific mean); Bolded numbers are added to denote nodes for ease of interpretation (see Table 2 for interpretation).

As shown in Table 1, the best-performing model includes the cumulative mean and deviations from that mean as predictors. This model has a cross-validated mean AUC value of 0.86 [standard error (s.e.) = 0.002], mean PPV of 0.74 (s.e. = 0.006), mean sensitivity of 0.81 (s.e. = 0.005), and mean specificity of 0.82 (s.e. = 0.006). Its corresponding tree is presented in Fig. 1. The simple model (previous-day scores) yielded a cross-validated mean AUC value of 0.82 (s.e. = 0.002), mean PPV of 0.69 (s.e. = 0.005), mean sensitivity of 0.79 (s.e. = 0.008), and mean specificity of 0.78 (s.e. = 0.006). Thus, the complex model resulted in a 4% improvement in accurately identifying suicidal ideation events, which is important to maximize in the context of suicide prevention^2^. The best-performing model (see Fig. 1) included a combination of cumulative mean and/or deviation from that mean for the following factors: ideation duration, hopelessness, burdensomeness, and self-efficacy. For instance, as shown in Fig. 1, one pathway leading to next-day suicidal ideation involves the following: 0.95 or lower cumulative mean of ideation duration, combined with 2.19 or lower cumulative mean of hopelessness, together with greater than 1.95 cumulative mean of burdensomeness and with −1.59 or lower change score of self-efficacy. Table 2 provides detailed information about interpreting this model's results, including specific thresholds in the decision tool shown in Fig. 1.

**Table 1.** Performance metrics for models predicting next-day suicidal ideation

	Model based on raw scores	Model based on cumulative mean and change scores
	Mean metric (s.e.)	Mean metric (s.e.)
AUC	0.82 (0.002)	0.86 (0.002)
PPV	0.69 (0.005)	0.74 (0.006)
Sensitivity	0.79 (0.008)	0.81 (0.005)
Specificity	0.78 (0.006)	0.82 (0.006)

**Table 2.** Interpretation of the best-performing model shown in Fig. 1

Node number (see Fig. 1)	Directions
1	a) Within-person cumulative mean of ideation duration greater than 0.95 = Move to node 3 b) Otherwise, move to node 2
2	a) Within-person cumulative mean of hopelessness is greater than 2.19 = No next day ideation b) Otherwise, move to node 4
3	a) Within-person change score of ideation duration is greater than −1 = Move to node 6 b) Otherwise, move to node 5
4	a) Within-person cumulative mean of burdensomeness is greater than 1.95 = Move to node 7 b) Otherwise, no next day ideation
5	a) Within-person cumulative mean of ideation duration is greater than 2.54 = Next day ideation b) Otherwise, no next day ideation
6	a) Within-person cumulative mean of burdensomeness is greater than 4.82 = Next day ideation b) Otherwise, move to node 8
7	a) Within-person change score of self-efficacy is greater than −1.59 = No next day ideation b) Otherwise, next day ideation
8	a) Within-person cumulative mean score of self-efficacy is greater than 8.86 = No next day ideation b) Otherwise, next day ideation

**Fig. 2.**
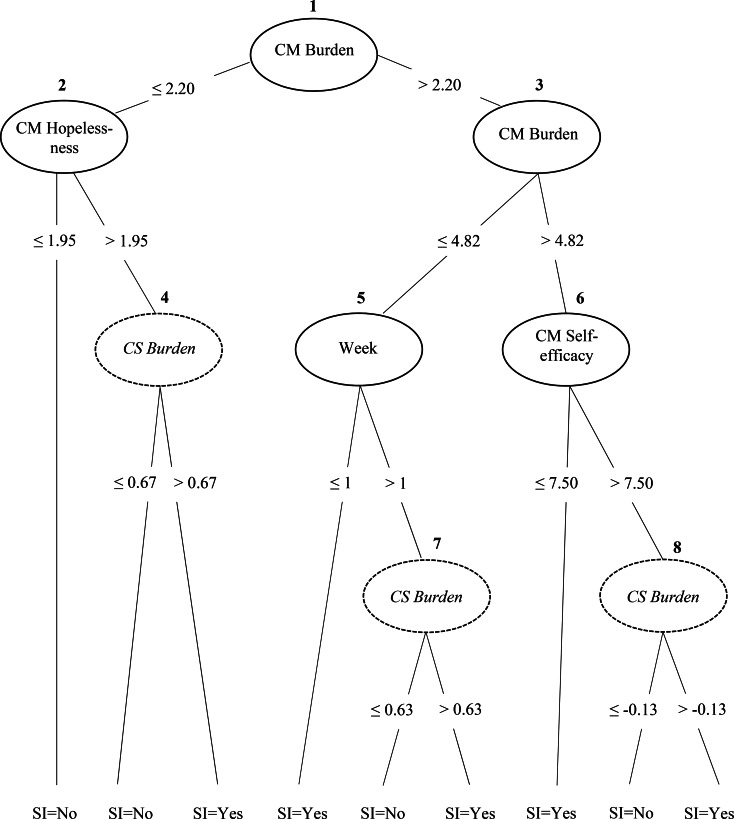
Prediction rule for next-day suicidal ideation excluding previous-day ideation duration. *Notes*: SI = Next-day suicidal ideation; CM = Cumulative person-specific mean; *CS* = Change score (deviation from the person-specific mean); Bolded numbers are added to denote nodes for ease of interpretation (see Supplementary Table S2 for interpretation).

As shown in Table 3, models without the duration of suicidal thoughts had acceptable – but relatively poorer – performance. The simple model without suicidal ideation duration was least predictive, with cross-validated mean AUC of 0.75 (s.e. = 0.005), mean PPV of 0.61 (s.e. = 0.006), mean sensitivity of 0.73 (s.e. = 0.009), and mean specificity of 0.71 (s.e. = 0.009). As shown in Fig. 2, the best-performing model that excluded suicidal ideation as a predictor (cross-validated mean AUC = 0.78) identified the following predictors for next-day ideation: burdensomeness, hopelessness, self-efficacy, and study week. Online Supplementary Table S2 provides information about interpreting this model's results, including specific thresholds in the decision tool, shown in Fig. 2.

**Table 3.** Performance metrics for models predicting next-day suicidal ideation excluding suicidal ideation duration as a predictor

	Model based on raw scores	Model based on cumulative mean and change scores
	Mean metric (s.e.)	Mean metric (s.e.)
AUC	0.75 (0.005)	0.78 (0.004)
PPV	0.61 (0.006)	0.60 (0.007)
Sensitivity	0.73 (0.009)	0.84 (0.012)
Specificity	0.71 (0.009)	0.64 (0.014)

## Discussion

In this study of suicidal adolescent inpatients, we applied machine learning methods (multi-level CARTs) to daily data collected over 4 weeks during the post-discharge period. The goal was to develop a decision tool that leverages these intensive longitudinal data to indicate each day whether the adolescent is likely to experience suicidal ideation on the next day. To our knowledge, this is the first study that used machine learning to identify different combinations of intensely sampled factors (and their specific thresholds) that predicted, with relatively high accuracy, the emergence of next-day suicidal ideation during the critical post-discharge period. The results further highlighted that such algorithmically optimized combinations hold promise for identifying specific states of vulnerability to near-term suicidal ideation that could, in turn, be used to guide *when* (i.e. the conditions in which) an intervention might be needed. The study's key findings and implications are discussed below.

First, we found that the best-performing CART model (AUC of 0.86) – which included time-varying cumulative means of risk factors together with deviations from the means – predicted next-day suicidal ideation with greater accuracy relative to a simpler model that included previous-day ratings of risk factors. The fact that prediction was improved by incorporating different features of risk factors (i.e. mean and deviation) is largely consistent with previous daily diary and EMA studies, where more complex models exhibited better prediction of suicide-related outcomes 2–4 weeks later (Czyz et al., 2020; Wang et al., 2021). Others have similarly shown that, in contrast to simpler (univariate) models, machine learning models accounting for complex combinations among predictors showed a stronger prediction of suicidal thoughts and attempts 3, 14, and 28 days later (Ribeiro et al., 2019). However, in contrast to previous machine learning studies of suicide-related outcomes assessed over longer intervals (Chen et al., 2020; de la Garza et al., 2021; Ribeiro et al., 2019; Walsh et al., 2017, 2018), CARTs in this study achieved relatively good predictive accuracy without using a large number of predictors (e.g. 50–3000 predictors in prior studies). Attending to the issues of parsimony is an important consideration for ultimately translating algorithms into clinical practice, particularly if such algorithms incorporate frequent assessments. In the current study, it was also notable that person-specific cumulative means of factors, in combination with more proximally occurring experiences (represented by deviations from the cumulative mean), emerged as being important in identifying next-day ideation. While somewhat intuitive (e.g. enduring hopelessness over prior days together with more recent increases in hopelessness make for a powerful combination), we are not aware of previous studies that examined how dynamically changing factors accumulate to influence near-term suicidal ideation or risk more broadly. It may be that examining short-term changes in risk and protective factors, together with their accumulation, provides a fuller picture of the processes impacting suicidal ideation in daily life. For example, previous studies have demonstrated that within-person changes in individual risk factors alone have limited prospective prediction of short-term suicidal thoughts (Ben-Zeev et al., 2012; Coppersmith et al., 2019; Kleiman et al., 2017).

Second, there were multiple pathways of risk factor combinations associated with next-day suicidal ideation, although the duration of suicidal thinking emerged as the top risk factor. This is in line with previous daily and EMA studies demonstrating that suicidal ideation tends to have the most consistent prospective link with suicidal thoughts (Coppersmith et al., 2019; Hallensleben et al., 2019; Kleiman et al., 2017). However, we also found that when excluding predictors based on ideation duration, next-day suicidal thoughts can still be adequately predicted based on other factors (e.g. one such pathway incorporated, at specific thresholds of each: cumulative hopelessness mean, cumulative burdensomeness mean, and change in self-efficacy score). In other words, the CART analysis identifies other pathways that could ‘flag’ next-day suicidal ideation. Beyond identifying *when* an intervention could potentially be provided, a promising application of identifying multiple pathways of near-term ideation risk could include the personalization of intervention content based on the unique constellation of risk factors (specific warning signs of near-term ideation). This is consistent with an expert consensus highlighting a range of warning signs related to suicide risk (Fowler, 2012; Rudd et al., 2006) as well as empirical evidence indicating that individuals exbibit different warning signs prior to attempting suicide (Bagge, Glenn, & Lee, 2013, 2017). Here, we show that near-term suicidal ideation is similarly multidetermined and thus could benefit from interventions addressing different risk pathways in daily life.

Finally, findings highlight that intensive assessment of prior ideation duration may not be needed to identify subsequent suicidal ideation risk. The more complex model without ideation duration yielded reasonably good predictive accuracy (AUC of 0.78). Previous studies have similarly found that models without suicidal ideation performed well in identifying the occurrence of suicidal thoughts and related crises within a few days to weeks (Czyz et al., 2020; Ribeiro et al., 2019). Excluding ideation duration in prediction models may offer practical advantages if a frequent assessment of suicidal thoughts is not feasible or if there are concerns that at-risk individuals may not disclose ideation if queried directly (Bernecker et al., 2019; Drum, Brownson, Burton Denmark, & Smith, 2009). However, as found in the current study, excluding ideation may lead to some tradeoffs in model performance (e.g. lower specificity). Additional practical considerations include sustaining response adherence (especially over longer periods of time) and minimizing response burden. Prediction models that are parsimonious and do not solely rely on self-report responses may be especially valuable. For example, real-time passive data not requiring direct input from individuals (e.g. sensors, geolocation, communication logs) may offer unique advantages for predicting near-term risk (Allen, Nelson, Brent, & Auerbach, 2019; Kleiman, Glenn, & Liu, 2019; Torous et al., 2018), although the utility of such data in predicting suicide-related outcomes, particularly in everyday life, still needs to be established to guide clinical decision-making.

## Study limitations and future directions

The study's findings should be considered in light of key limitations. Adolescents in this study were primarily White and were recruited from a single inpatient unit, which might limit generalizability. The findings should be replicated in other samples. While the risk factors included in analyses were selected based on clinical and theoretical considerations, they do not offer an exhaustive list of constructs that can be explored. Future research could consider additional risk and protective factors along with additional variable features. Given limitations inherent in self-report data (e.g. social desirability, recall bias, response burden), additional research should also consider examining predictors based on other types of assessment, such as passively collected data. Future extensions of this work, particularly involving large samples, could examine the extent to which near-term risk algorithms incorporating intensive longitudinal data may vary as a function of participants' initial characteristics (e.g. suicide attempt history) as well as investigate their predictive performance beyond the near-term window (ideation outcome defined as 2 days later, 3 days later, etc.). In addition to detecting its near-term presence, future extensions of this work could also consider predicting proximal change in suicidal ideation (e.g. continued absence, continued presence, decline, or emergence). Moreover, while we tested the stability of results using repeated 5-fold cross-validation, replicating these findings in an independent sample would offer the most rigorous validation approach. While beyond the scope of this study, future research is also needed to determine the practical utility of such prediction algorithms in informing intervention development, including how to intervene when individuals are vulnerable to next-day suicidal thoughts and whether individuals at risk for suicide are receptive to such real-time interventions.

## Conclusions

With the goal of developing a decision tool that leverages intensive longitudinal data to identify near-term suicidal ideation in a high-risk clinical sample, this study advances prior research focusing on the prediction of short-term suicide risk. Among recently discharged adolescents, we applied a series of multi-level CART models incorporating dynamically changing risk and protective factors to predict next-day suicidal thoughts. While simpler models that included previous-day ratings of risk and protective factors yielded adequate predictive accuracy, CART models achieved improved performance when including as predictors both the cumulative means of factors over prior days and deviations from these means. These results illustrate that intensive longitudinal data could guide the delivery of real-time interventions for suicide prevention by identifying specific states of vulnerability to near-term suicidal thoughts. Additional research is needed to validate results in independent samples as well as build on this work by improving generalizability to other populations and settings, incorporating different risk factors and data collection methods, as well as by attending to practical issues that could impact the translation of prediction algorithms into clinical settings.

## Footnotes

† The notes appear after the main text.

## Notes

1 The results did not differ when the pilot intervention conditions were controlled for in the models. Thus, intervention conditions were removed from the final models.

2 In supplemental analyses, we considered whether the best-performing model could be further improved by adding previous-day scores (i.e. including all three features: cumulative means, deviations, and previous-day scores). Notably, this model (cross validated AUC = 0.85; PPV = 0.74; Sensitivity = 0.76; specificity = 0.81) did not yield improved prediction, suggesting previous-day scores did not contribute additional information to the best-performing model.

## Supplementary material

For supplementary material accompanying this paper visit https://doi.org/10.1017/S0033291721005006. click here to view supplementary material
