# Supplementary material for: Predicting short-term suicidal thoughts in adolescents using machine learning: developing decision tools to identify daily level risk after hospitalization
Source: Psychol Med. 2021 Dec 9;53(7):2982–91. doi: 10.1017/S0033291721005006 (PMC9814182; doi:10.1017/S0033291721005006)
Supplement: Supplementary file 1 [file S0033291721005006sup001.docx]

| Supplemental Table 1. Descriptive statistics |  |  |
| --- | --- | --- |
| *Predictors* | Mean (SE) | ICC |
| Duration of Suicide Ideation (0-5) | 0.97 (0.12) | 0.59 |
| Hopelessness (1-4) | 2.01 (0.08) | 0.58 |
| Connected to family (1-7) | 4.66 (0.16) | 0.59 |
| Connected to friends (1-7) | 4.80 (0.14) | 0.44 |
| Burdensomeness (1-7) | 2.89 (0.17) | 0.62 |
| Agitation (1-7) | 3.02 (0.20) | 0.62 |
| Worry (1-7) | 3.45 (0.16) | 0.48 |
| Rumination (1-7) | 3.16 (0.15) | 0.47 |
| Self-efficacy (0-10) | 7.87 (0.24) | 0.67 |
| Psychological pain (1-5) | 2.14 (0.10) | 0.50 |
|  |  |  |
| *Outcome* | N (%) |  |
| Next-day suicidal ideation | 592 (37.9) |  |

^a^ SE: Standard Error; ICC: Intraclass Correlation Coefficient

Supplemental Table 2. Interpretation of model shown in Figure 2

| **Node number (see Figure 2)** | **Directions** |
| --- | --- |
| **1** | a) Within-person cumulative mean of burdensomeness is greater than 2.20 = Move to node 3  b) Otherwise, move to node 2 |
| **2** | a) Within-person cumulative mean of hopelessness is greater than 1.95 = Move to node 4  b) Otherwise, no next day ideation |
| **3** | a) Within-person cumulative mean of burdensomeness is greater than 4.82 = Move to node 6  b) Otherwise, move to node 5 |
| **4** | a) Within-person change score of burdensomeness is greater than 0.67 = Next day ideation  b) Otherwise, no next day ideation |
| **5** | a) Week is greater than 1 = Move to node 7  b) Otherwise, next day ideation |
| **6** | a) Within-person cumulative mean of self-efficacy is greater than 7.50 = Move to node 8  b) Otherwise, next day ideation |
| **7** | a) Within-person change score of burdensomeness is greater than 0.63 = Next day ideation  b) Otherwise, no next day ideation |
| **8** | a) Within-person change score of burdensomeness is greater than -0.13 = Next day ideation  b) Otherwise, no next day ideation |
